# The safety and immunogenicity of two novel live attenuated monovalent (serotype 2) oral poliovirus vaccines in healthy adults: a double-blind, single-centre phase 1 study

**DOI:** 10.1016/S0140-6736(19)31279-6

**Published:** 2019-07-13

**Authors:** Pierre Van Damme, Ilse De Coster, Ananda S Bandyopadhyay, Hilde Revets, Kanchanamala Withanage, Philippe De Smedt, Leen Suykens, M Steven Oberste, William C Weldon, Sue Ann Costa-Clemens, Ralf Clemens, John Modlin, Amy J Weiner, Andrew J Macadam, Raul Andino, Olen M Kew, Jennifer L Konopka-Anstadt, Cara C Burns, John Konz, Rahnuma Wahid, Christopher Gast

**Affiliations:** a Centre for the Evaluation of Vaccination, Vaccine and Infectious Disease Institute, University of Antwerp, Antwerp, Belgium; b Bill & Melinda Gates Foundation, Seattle, WA, USA; c Centers for Disease Control and Prevention, Atlanta, GA, USA; d Institute for Global Health, University of Siena, Siena, Italy; e Global Research in Infectious Diseases, Rio de Janeiro, Brazil; f National Institute for Biological Standards and Control, Ridge, UK; g Department of Microbiology and Immunology, University of California San Francisco, San Francisco, CA, USA; h Center for Vaccine Innovation and Access, PATH, Seattle, WA, USA

## Abstract

**Background:**

Use of oral live-attenuated polio vaccines (OPV), and injected inactivated polio vaccines (IPV) has almost achieved global eradication of wild polio viruses. To address the goals of achieving and maintaining global eradication and minimising the risk of outbreaks of vaccine-derived polioviruses, we tested novel monovalent oral type-2 poliovirus (OPV2) vaccine candidates that are genetically more stable than existing OPVs, with a lower risk of reversion to neurovirulence. Our study represents the first in-human testing of these two novel OPV2 candidates. We aimed to evaluate the safety and immunogenicity of these vaccines, the presence and extent of faecal shedding, and the neurovirulence of shed virus.

**Methods:**

In this double-blind, single-centre phase 1 trial, we isolated participants in a purpose-built containment facility at the University of Antwerp Hospital (Antwerp, Belgium), to minimise the risk of environmental release of the novel OPV2 candidates. Participants, who were recruited by local advertising, were adults (aged 18–50 years) in good health who had previously been vaccinated with IPV, and who would not have any contact with immunosuppressed or unvaccinated people for the duration of faecal shedding at the end of the study. The first participant randomly chose an envelope containing the name of a vaccine candidate, and this determined their allocation; the next 14 participants to be enrolled in the study were sequentially allocated to this group and received the same vaccine. The subsequent 15 participants enrolled after this group were allocated to receive the other vaccine. Participants and the study staff were masked to vaccine groups until the end of the study period. Participants each received a single dose of one vaccine candidate (candidate 1, S2/cre5/S15domV/rec1/hifi3; or candidate 2, S2/S15domV/CpG40), and they were monitored for adverse events, immune responses, and faecal shedding of the vaccine virus for 28 days. Shed virus isolates were tested for the genetic stability of attenuation. The primary outcomes were the incidence and type of serious and severe adverse events, the proportion of participants showing viral shedding in their stools, the time to cessation of viral shedding, the cell culture infective dose of shed virus in virus-positive stools, and a combined index of the prevalence, duration, and quantity of viral shedding in all participants. This study is registered with EudraCT, number 2017-000908-21 and ClinicalTrials.gov, number NCT03430349.

**Findings:**

Between May 22 and Aug 22, 2017, 48 volunteers were screened, of whom 15 (31%) volunteers were excluded for reasons relating to the inclusion or exclusion criteria, three (6%) volunteers were not treated because of restrictions to the number of participants in each group, and 30 (63%) volunteers were sequentially allocated to groups (15 participants per group). Both novel OPV2 candidates were immunogenic and increased the median blood titre of serum neutralising antibodies; all participants were seroprotected after vaccination. Both candidates had acceptable tolerability, and no serious adverse events occurred during the study. However, severe events were reported in six (40%) participants receiving candidate 1 (eight events) and nine (60%) participants receiving candidate 2 (12 events); most of these events were increased blood creatinine phosphokinase but were not accompanied by clinical signs or symptoms. Vaccine virus was detected in the stools of 15 (100%) participants receiving vaccine candidate 1 and 13 (87%) participants receiving vaccine candidate 2. Vaccine poliovirus shedding stopped at a median of 23 days (IQR 15–36) after candidate 1 administration and 12 days (1–23) after candidate 2 administration. Total shedding, described by the estimated median shedding index (50% cell culture infective dose/g), was observed to be greater with candidate 1 than candidate 2 across all participants (2·8 [95% CI 1·8–3·5] *vs* 1·0 [0·7–1·6]). Reversion to neurovirulence, assessed as paralysis of transgenic mice, was low in isolates from those vaccinated with both candidates, and sequencing of shed virus indicated that there was no loss of attenuation in domain V of the 5ʹ-untranslated region, the primary site of reversion in Sabin OPV.

**Interpretation:**

We found that the novel OPV2 candidates were safe and immunogenic in IPV-immunised adults, and our data support the further development of these vaccines to potentially be used for maintaining global eradication of neurovirulent type-2 polioviruses.

**Funding:**

Bill & Melinda Gates Foundation.

## Introduction

The world is on the threshold of achieving the 1988 World Health Assembly's goal to eradicate poliovirus. Eradication of wild type-2 polioviruses was confirmed in September, 2015, leading the WHO Strategic Advisory Group of Experts to recommend global cessation of use of vaccines containing live Sabin type-2 poliovirus.^1^, ^2^ By May, 2016, trivalent oral poliovirus vaccines (OPV) were replaced with bivalent OPV that only contained poliovirus type 1 and type 3, and they were supplemented with at least one dose of trivalent inactivated poliovirus vaccine (IPV) in an unprecedented globally synchronised effort.^3^ Although OPV use has interrupted poliovirus transmission in most of the world, on rare occasions, live-attenuated vaccine polioviruses can produce a neurological disease, termed vaccine-associated paralytic poliomyelitis, or they can acquire neurovirulence and transmissibility, creating the infectious circulating vaccine-derived polioviruses (cVDPVs).^4^, ^5^ For instance, in 2018, type-1 cVDPV outbreaks in Papua New Guinea^6^ and Indonesia and other type-2 cVDPV outbreaks^7^ occurred.

Since global cessation of OPV2 use in May, 2016, distinct circulating vaccine-derived type-2 poliovirus (cVDPV2) outbreaks have occurred in seven countries, with more than 150 cases of vaccine-derived poliovirus reported. cVDPVs have been designated a Public Health Emergency of International Concern, for which the only effective control is use of stockpiled monovalent OPV2. Use of this vaccine carries the inherent risk of seeding new cVDPVs, particularly with waning mucosal immunity of the population following OPV2 cessation.^8^, ^9^ IPV use cannot generate cVDPVs but these vaccines do not induce primary intestinal mucosal immunity, so IPV use is ineffective in interrupting transmission in settings where transmission is predominantly via the faecal–oral route.^10^, ^11^ Therefore novel OPV2 candidates have been developed with improved genetic stability, which decreases the likelihood of reversion to neurovirulence, thereby minimising the risk of generating new cVDPVs.^11^

**Research in context**

**Evidence before this study**

Our study is the first in-human investigation of live-attenuated novel oral type-2 poliovirus vaccine candidates that assesses their safety, tolerability, immunogenicity, and stability of attenuation after intestinal passage, and it represents the first initiative to develop a new oral polio vaccine in more than 50 years. A formal literature search was not performed.

**Added value of this study**

We found that the novel OPV2 vaccine candidates are safe, well tolerated, and sufficiently immunogenic in the population tested. We also found that both vaccine candidates show a substantially lower incidence of reversion of the attenuations than Sabin oral type-2 polio vaccines (OPV2), and thus present a lower risk of reacquiring neurovirulent properties.

**Implications of all the available evidence**

Despite the global withdrawal of live type-2 poliovirus vaccines from routine use since May, 2016, subsequent outbreaks of circulating vaccine-derived type-2 polioviruses that have spread transnationally across Africa highlight the importance of having stockpiles of monovalent OPV2 vaccines available; such vaccines are the only effective means to prevent transmission and control these outbreaks. Because most circulating vaccine-derived polioviruses originate from the Sabin OPV2 vaccines, it is important that these vaccines are replaced with more genetically stable vaccines, such as the two candidates that we investigate, to complete eradication of poliomyelitis. The novel clinical evidence generated by our phase 1 clinical trial, including the unique information on genetic stability and neurovirulence potential, can be used to accelerate the next phase of clinical development and inform global policy making regarding replacement of the global stockpiles of monovalent Sabin OPV2 with novel OPV2, to respond to ongoing and future outbreaks of circulating vaccine-derived type-2 poliovirus.

Given the unique context of certified wild type-2 poliovirus eradication, OPV2 withdrawal, and WHO GAPIII containment guidelines,^12^ additional measures were needed to study these novel OPV2 candidates in humans. In the first clinical trial of a new polio vaccine in more than 50 years, we report the first in-human phase 1 trial of two novel OPV2 candidates (S2/cre5/S15domV/rec1/hifi3 and S2/S15domV/CpG40). Our primary aims were to evaluate the safety and shedding of these vaccines in IPV-immunised adults in a purpose-built, contained research unit designed to prevent release into the general population, as a further step toward the permanent global eradication of poliomyelitis.

## Methods

### Study design and participants

In this double-blind, single-centre phase 1 trial, we used local advertising to recruit volunteers at the University of Antwerp Hospital (Antwerp, Belgium). In addition to the restrictions imposed by GAPIII^12^ that apply to all type-2 polioviruses, the novelty of the genetically modified novel OPV2 viruses necessitated performing the study in a fully contained, purpose-built facility (which we named Poliopolis), to avoid any accidental environmental release by ensuring that all biological samples that could potentially contain vaccine virus could be captured and contained for subsequent decontamination.

Screening for inclusion included a medical examination, laboratory testing (serology, chemistry, coagulation, and haematology), and interviews by two psychologists to assess the volunteers' mental fitness to cope with the confinement and restrictions of containment for 28 days and their compatibility with each other. Eligible volunteers were healthy men or women (aged 18–50 years), with complete IPV-only polio vaccination histories. Inclusion criteria included a willingness to adhere to all prohibitions and restrictions necessary for full containment for the study duration, no intended travel to polio-endemic countries or the Netherlands (because of low vaccination coverage in the so-called Bible belt), and no professional food handling activity or household or professional contact with immunosuppressed individuals or people without a full poliovirus vaccination (such as infants under 6 months of age). These restrictions were to be enforced until viral shedding ceased after participants left Poliopolis. Principal exclusion criteria included any condition that the investigator believed could compromise the participant's wellbeing, any gastrointestinal condition (eg, Crohn's disease or ulcerative colitis), receipt of immunosuppressive medication within 6 months preceding the start of the study, previous receipt of OPV, any polio vaccination within 12 months of the start of the study, or any other vaccinations within 28 days of the study or planned within the study period. Women of childbearing age were required to have a negative urine pregnancy test on day 0, not to be breastfeeding, and to use an approved contraceptive method until 3 months after vaccine administration. Volunteers were enrolled sequentially in two groups, with each group receiving one of the two vaccine candidates, to avoid cross-contamination, after which they were confined to Poliopolis for 28 days, with further monitoring until end of shedding.

All participants provided written informed consent at enrolment, and the study was overseen by an independent Data and Safety Monitoring Board. Ethical approval was received from university and hospital institutional review boards and the study was done according to prevailing Declaration of Helsinki and ICH Good Clinical Practice guidelines. The study protocol was reviewed by the US Centers for Disease Control and Prevention (CDC) Human Research Protection Office and determined that CDC was not-engaged.

### Randomisation and masking

After a screening visit, in which baseline blood and stool samples were collected, we enrolled the first three volunteers in the first group sequentially. Participants and study staff assessing shedding were masked to vaccine group allocations until the end of the study period by labelling of the vaccines with the letters A and B (labelling done by CSM, Belgium). The first participant opened one of two envelopes to select vaccine A or B for that group and received the corresponding vaccine (candidate 2). If no serious or severe adverse events were reported within 48 h, volunteer 2 was enrolled and vaccinated, and this method was repeated for volunteer 3. The Data and Safety Monitoring Board assessed safety data from these first three participants within 24 h, before approving enrolment of the remaining 12 volunteers for that group. The subsequent 15 participants enrolled after this group were allocated to receive the other vaccine.

### Vaccines

The novel OPV2 candidates are live-attenuated serotype-2 polioviruses that were derived from a modified Sabin type-2 infectious cDNA clone and propagated in Vero cells; candidate 1 (S2/cre5/S15domV/rec1/hifi3) was given to the second group to be sequentially allocated (participants 16 to 30) and candidate 2 (S2/S15domV/CpG40) was given to the first 15 participants allocated to groups. We modified the viral nucleotide sequences in part of the 5ʹ-untranslated region in both candidates, to improve the genetic stability of this major attenuating determinant of Sabin type-2. In candidate 1, this alteration was augmented with two modifications in the polymerase 3D to further improve stability of the attenuation, and relocation of a key replication element from the 2C coding region to the 5ʹ-untranslated region, to inhibit recombination. In candidate 2, silent non-coding modifications engineered within the capsid (VP1–4) were designed to reduce replicative fitness and, potentially, to improve stability of the attenuated phenotype while also reducing transmission. Full details of the genetic modifications and preclinical testing of both candidates will be published separately.

Clinical trial lots of both novel OPV2 candidates underwent manufacturing release testing, including standard WHO monkey neurovirulence testing, and vaccine formulation by use of methods employed for current Sabin-based OPV products by P T Bio Farma (Bandung, Indonesia).^13^ To establish safety in our phase 1 study, a high dose of approximately 10^6^ 50% cell culture infectious dose units (CCID_50_) was administered orally as six drops (totalling 0·3 mL), which were given with a supplied dropper to guarantee the dose.

### Procedures

After vaccination on day 0, we monitored volunteers for adverse events, and we assessed them with safety laboratory tests, including evaluations of viral shedding in stools, nasopharyngeal secretions, and humoral immunity. Containment was intended to last until three consecutive stool samples were virus-free, determined by PCR, for all participants in a group or until the 28th day after vaccination, whichever occurred first, meaning that the first three volunteers remained in the containment facility for 35 (first participant), 33 (second participant), and 31 days (third participant). If shedding persisted after 28 days, participants were allowed to leave but they were requested to remain in Belgium and to continue providing stool samples in an ambulatory manner by use of provided chemical toilets (with a stool storage capacity of 3–4 days) and mandatory containers for infectious waste disposal. A final safety follow-up call (for those no longer shedding) or visit (for those still shedding) was made 42 days after vaccine administration. After completion of the study by the first group and cleaning and decontamination of the facility, this procedure was repeated in an identical manner for the second group, who were given novel OPV2 candidate 1.

During containment, we gave participants physical examinations daily, starting on day 0, and particularly on days 7 and 28, and further examinations were made as required after presentation of symptoms of adverse events. We took blood samples for laboratory assessments at screening and on days 7, 14, and 28 for standard haematology analyses and blood chemistry measurements; we used the Common Terminology Criteria for Adverse Events version 4.03 (toxicity grades) to record clinical events or the US Food and Drug Administration manual for clinical laboratory measurements. A medical team consisting of four doctors conducted daily consultations with all participants throughout the containment period, and they questioned participants on any mental or physical issues.

Adverse events were solicited from the participants by the medical team during the 7 days after vaccination. Solicited events comprised signs and symptoms that were reported with a predefined checklist (headache, fatigue, myalgia, arthralgia, paraesthesia, anaesthesia, paralysis, nausea, vomiting, diarrhoea and abdominal pain) in a diary card. Unsolicited events comprised other signs and symptoms that participants recorded in their diary card. Adverse events were graded as mild (easily tolerated), moderate (sufficiently discomforting to interfere with normal everyday activities), or severe (preventing normal everyday activities), and they were also assessed by the investigator for causality (unrelated, unlikely, possibly, or probably related to the vaccination). Oral temperature was also measured by participants during days 0–7; body temperatures of 37·5°C or more were defined as a fever, and temperatures of more than 39·0°C were defined as a severe fever.

Strict procedures were imposed to collect daily stool samples, which were partly processed on-site to prepare for shipping at regular intervals to the CDC (Atlanta, GA, USA). Samples were stored at −20°C until analysis. We detected the type-2 poliovirus genome with a Sabin multiplex real-time RT-PCR assay of total nucleic acid extracted from stool suspensions (50% weight to volume in cell culture medium).^14^ Nucleic acid extraction was done with a Thermo Fisher Scientific KingFisher Flex 96-deep-well analyser.^15^ Before extraction, stool suspensions were spiked with an extraction control (Qβ bacteriophage; Attostar) and detected with Qβ-specific real-time RT-PCR; stool suspensions with a negative extraction control (Ct>40, indicating inefficient extraction) were re-extracted. In samples that were positive for type-2 poliovirus, infectious virus was measured as CCID_50_ per g of stool by use of a modification of the WHO cell sensitivity assay, as described previously.^16^ Nasopharyngeal swabs that were obtained from each participant on days 0, 3, 7, and the final day of containment were processed and shipped to the CDC laboratory, where they were stored at −20°C until real-time RT-PCR evaluation for the presence of poliovirus type-2 with the same procedure as the stool suspensions.

Humoral immunogenicity was assessed as poliovirus type-2-specific serum neutralising antibodies at days 0 and 28 with a standard protocol.^16^ We calculated the median and geometric mean titres, seroprotection (the proportion of the groups with poliovirus type-2-specific antibody reciprocal titres ≥8), and seroconversion at day 28 from these samples. Seroconversion was defined as a change from seronegative to seropositive (poliovirus type-2- specific antibody reciprocal titres ≥8) or, in seropositive participants, an antibody titre increase of at least four-fold more than baseline.

The specimen tested for neurovirulence, the exploratory endpoint specimen, was the last stool sample provided by each participant that had adequate concentrations of virus for the neurovirulence and deep sequencing assays (≥4 log_10_[CCID_50_/g]). The WHO poliovirus receptor transgenic mouse (Tg-PVR21) neurovirulence test that characterises the potential for neurovirulence of shed virus^17^ was modified and we used this modified test to evaluate exploratory endpoint specimen samples. Detected polioviruses in exploratory endpoint specimen samples were amplified in HEp2-C cells for 3 days at 33°C to achieve sufficient virus for each mouse inoculation. Briefly, for each exploratory endpoint specimen ten Tg-PVR21 mice were each administered intraspinal inoculations of 4 log_10_(CCID_50_) amplified virus in 5 μL volumes, and each exploratory endpoint specimen was tested in triplicate. Candidate 1 and 2 clinical trial bulk preparations of viruses were also tested at the 4 log_10_(CCID_50_) dose. As controls ten mice were each inoculated with SO+2/II at 5·0 log_10_(CCID_50_) and 6·0 log_10_(CCID_50_) doses^17^ and a sample of shed Sabin 2 virus collected 7 days after vaccination with monovalent OPV2 in a previous clinical trial.^18^ Inoculated mice were monitored for paralysis for a 14-day observation period as per established guidance.^17^ At the end of the observation period, a final outcome of paralysed or non-paralysed was assigned to each mouse, to determine the paralysis frequency per exploratory endpoint specimen.

We also examined exploratory endpoint specimens by deep sequencing, to assess their genetic stability by demonstrating the retention of key genetic regions engineered in the vaccine candidates. Deep sequencing was performed on the cell culture-amplified virus and on viral RNA isolated directly from a 10% suspension of the exploratory endpoint specimen of each participant, to assess retention of these genetic modifications. Viral RNA was isolated from amplified viral stock from HEp2-C cells or stool by use of a QIAamp Viral RNA Mini kit (Qiagen), followed by cDNA synthesis and full-length poliovirus genome amplification (KOD Xtreme Hot Start DNA Polymerase kit; Millipore). We did tagmentation and library preparation with the Nextera XT kit (Illumina), followed by 300-cycle paired-end sequencing with MiSeq reagent kit version 3 reagents on a MiSeq sequencer and MiSeq analysis software version 1.8.46 (all Illumina) to generate FASTQ files.^19^

### Outcomes

The primary outcomes, which were assessed in both groups, were the safety of the novel OPV2 candidates by assessment of the incidence and type of serious and severe adverse events, the proportion of participants showing viral shedding in their stools, the time to cessation of viral shedding, the cell culture infective dose of shed virus in virus-positive stools, and a combined index of the prevalence, duration, and quantity of viral shedding in all participants.

The secondary outcomes, assessed in both groups, were the incidence, severity, and type of adverse events (solicited and unsolicited, for the first 7 days and throughout the study period), deviations from reference laboratory results, the median titres of type-2 poliovirus antibodies in participants' serum at days 0 and 28, the proportion of participants with seroprotection at days 0 and 28, the proportion of participants showing seroconversion at day 28, and the neurovirulence of shed virus from exploratory endpoint specimens in a mouse model.

The exploratory outcomes were the geometric mean titre of type-2 poliovirus in all participants' serum at days 0 and 28, the genetic stability of shed virus in a subset of stool samples, and nasopharyngeal viral shedding in swabs from all participants.

### Statistical analysis

The sample size of 15 participants per group was considered reasonable and sufficient for a first-in-human contained study of investigational vaccines, as agreed by the stakeholders from the The Global Polio Eradication Initiative, to gain a preliminary assessment of safety and the incidence, quantity, and characteristics of shed virus. There was no hypothesis testing, since all analyses were descriptive.

For binary variables, which included safety endpoints, seropositivity and seroconversion, viral shedding, and mouse paralysis, numbers and percentages are shown with two-sided 95% CIs, which were computed by the exact or score method. Antibody geometric mean titres were calculated with 95% CIs with asymptotic methods on the log scale, and they were back-transformed with the upper limit of quantitation (1448 or 10·5 log_2_) as an observed value where necessary. Median titres and log_10_[CCID_50_/g] of shed virus in stools were shown with bootstrap-based 95% CIs with 10 000 replicates. As described previously,^20^ a viral shedding index was calculated as the average of log_10_[CCID_50_/g] of samples collected 7, 14, 21, and 28 days after vaccine administration, with the lower limit of quantitation (2·75 log_10_) as an observed value, and with real-time RT-PCR-negative values contributing 0 to the mean. Missing values (from missing samples on specific study days) were replaced with values from the days before or after or the average of these two values, as necessary. SAS version 9.3 was used for analyses. This study is registered with EudraCT, number 2017-000908-21 and ClinicalTrials.gov, number NCT03430349.

### Role of the funding source

Three authors of this report (ASB, JM, AJW) are employees of the study funder. ASB was involved in study design, data analysis, data interpretation, and writing of the report. JM and AJW contributed to the study design and reviewed the manuscript. The funder of the study had no role in data collection. The corresponding author had full access to all the data in the study and had final responsibility for the decision to submit for publication.

## Results

**Figure 1:**
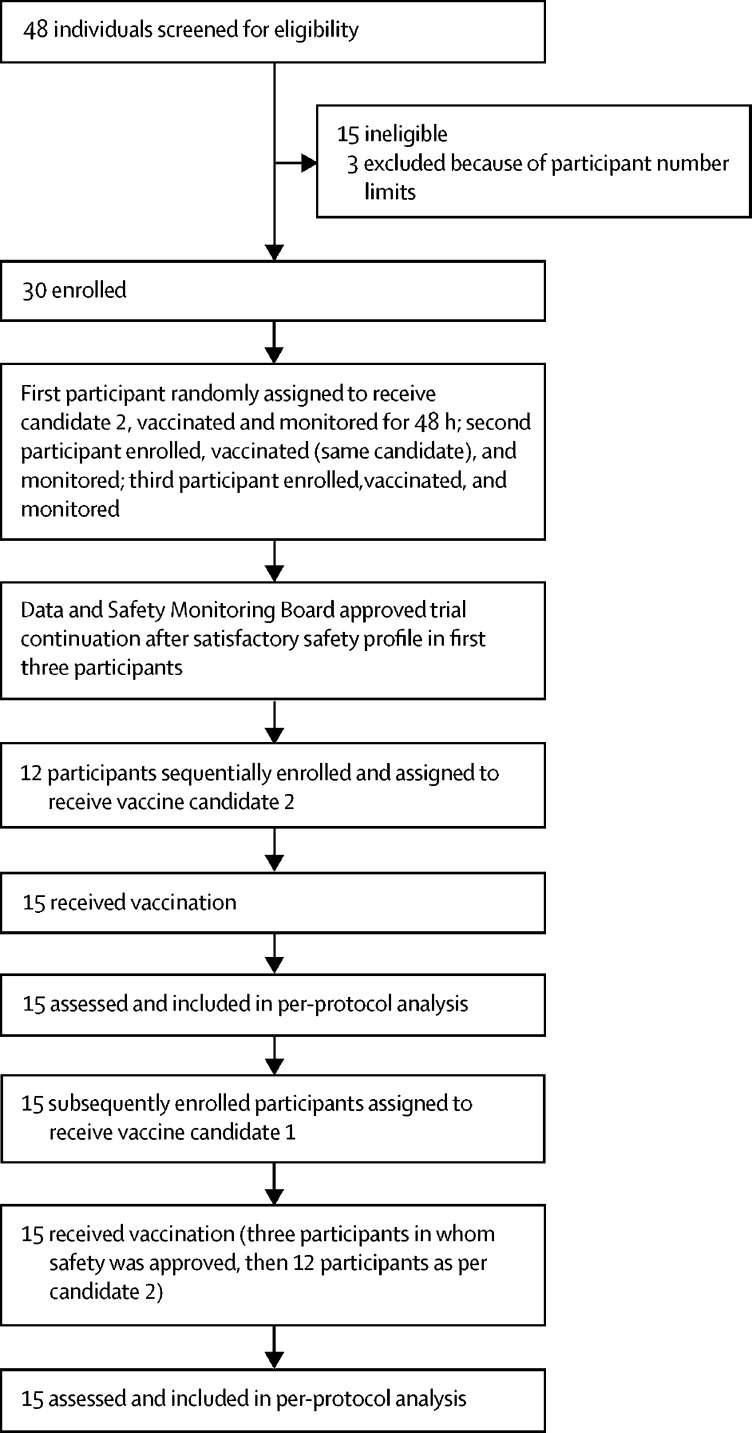
Trial profile

Between May 22 and Aug 22, 2017, 48 volunteers were screened, of whom 15 (31%) volunteers were excluded for reasons relating to the inclusion or exclusion criteria (figure 1). The main reasons for not being enrolled were psychological incompatibility with other selected participants and laboratory test abnormalities (eg, abnormal blood cell counts or liver enzyme concentrations). Three (6%) volunteers were not treated because of restrictions to the number of participants in each group, but they were retained as back-ups in case of early withdrawal by any participants who were enrolled and later excluded.

Participants were predominantly male (25 [83%] of 30 participants), with a mean age of 32·8 years, ranging from 21 to 50 years (table 1). Both groups were similar in terms of sex and age distribution. Vaccination records showed that 24 (80%) participants had received at least six IPV vaccinations, and 28 (93%) participants had received at least five IPV vaccinations; no participants had received OPV previously. There were no early terminations from the study and all participants fully complied with all procedures and sampling requests.

**Table 1:** Study group demographics

	**Candidate 1 (n=15)**	**Candidate 2 (n=15)**	**Total (n=30)**
**Sex, n (%)**			
Male	13 (87%)	12 (80%)	25 (83%)
Female	2 (13%)	3 (20%)	5 (17%)
**Age, years**			
Mean (SD)	31·1 (7·7)	33·5 (10·9)	32·3 (9·4)
Minimum–maximum	21–50	21–49	21–50
**Race, n (%)**			
White	14 (93%)	14 (93%)	28 (93%)
Asian	0	0	0
Black	1 (7%)	0	1 (3%)
Other	0	1 (7%)	1 (3%)
**Height, cm**			
Mean (SD)	182·2 (8·7)	180·0 (10·1)	181·1 (9·3)
Minimum–maximum	163·0–193·0	160·0–198·5	160·0–198·5
**Weight, kg**			
Mean (SD)	79·1 (15·6)	79·7 (11·8)	79·4 (13·6)
Minimum–maximum	53·6–114·1	64·9–95·4	53·6–114·1
**Body-mass index, kg/m^2^**			
Mean (SD)	23·7 (3·9)	24·7 (3·7)	24·2 (3·7)
Minimum–maximum	19·0–31·3	19·0–30·7	19·0–31·3

There were no serious adverse events (table 2). 15 participants (six [40%] participants receiving candidate 1 and nine [60%] participants receiving candidate 2) presented with 20 severe adverse events (eight events with candidate 1 and 12 events with candidate 2). Of these severe adverse events, seven events (in six participants receiving candidate 1) and ten events (in nine participants receiving candidate 2) were judged to possibly be related to the vaccine. Most of these severe adverse events were transient increases in blood creatinine phosphokinase (six events with candidate 1 and nine events with candidate 2), and the other two events were increased aspartate aminotransferase concentrations (one event with each candidate vaccine); none of these transient increases was associated with clinical signs or symptoms. Other severe adverse events (which were considered to be unlikely to be associated with the vaccine) were individual episodes of diarrhoea and gastroenteritis in individuals receiving candidate 2, and severe headache in a participant receiving candidate 1. Most severe adverse events resolved spontaneously or with standard treatments within the study period.

**Table 2:** Adverse events in the total vaccinated population

		**Candidate 1**		**Candidate 2**		**Total**	
		Number of participants (% of n=15)	Number of events	Number of participants (% of n=15)	Number of events	Number of participants (% of n=30)	Number of events
≥1 serious adverse event		0	0	0	0	0	0
≥1 severe adverse event		6 (40%)	8	9 (60%)	12	15 (50%)	20
	Probable	0	0	0	0	0	0
	Possible	6 (40%)	7	9 (60%)	10	15 (50%)	17
	Unlikely	0	1	0	2	0 (3%)	3
	Unrelated	0	0	0	0	0	0
Adverse events that led to study withdrawal		0	0	0	0	0	0
≥1 adverse event		15 (100%)	103	15 (100%)	84	30 (100%)	187
	Any solicited	13 (87%)	31	9 (60%)	18	22 (73%)	49
	Mild solicited	8 (53%)	25	8 (53%)	17	16 (53%)	42
	Moderate solicited	5 (33%)	6	1 (7%)	1	6 (20%)	7
	Severe solicited	0	0	0	0	0	0
	Any unsolicited	15 (100%)	72	15 (100%)	66	30 (100%)	138
	Mild unsolicited	3 (20%)	51	3 (20%)	37	6 (20%)	88
	Moderate unsolicited	6 (40%)	13	3 (20%)	17	9 (30%)	30
	Severe unsolicited	6 (40%)	8	9 (60%)	12	15 (50%)	20

Each participant was counted only once in each category, under the maximum causality or severity. Both solicited and unsolicited events are included, unless otherwise noted. Serious adverse events are medical events that are life-threatening, require admission to hospital, or result in notable incapacity of the individual. Severe adverse events are medical events that prevent normal everyday activities, and this category did not include serious adverse events. Solicited events comprised signs and symptoms that were reported by use of a predefined checklist in a diary card; or a fever, as determined by the participant's measurement of their body temperature. Unsolicited events comprised other signs and symptoms that participants recorded in their diary card. Participants graded their adverse events from mild to severe.

**Figure 2:**
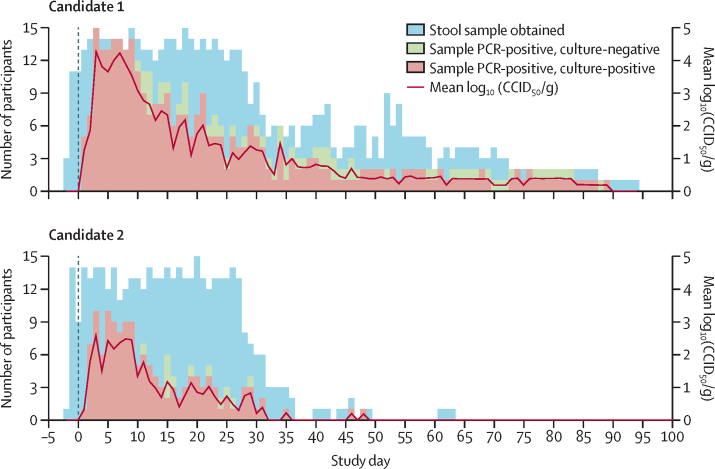
Viral shedding of the two novel oral type-2 poliovirus vaccine candidates Data are the number of PCR-positive stool samples and the log_10_(CCID_50_/g) after administration on day 0. Participants who had ceased shedding were included, at a value of 0, in the computation of the mean log_10_(CCID_50_/g). CCID_50_=50% cell culture infectious dose.

Viral RNA was detected in stool samples from most participants within a few days of administration, and was eventually positively identified in 15 (100%) participants who received candidate 1 and 13 (87%) participants receiving candidate 2. There was a gradual decrease in the number of participants shedding virus (as determined by RNA-positive stool samples) during the study period (figure 2). Observed shedding ceased (judged as three consecutive real-time RT-PCR-negative samples) and then resumed in three (20%) participants in each group, for a further 3–6 days. The shedding duration was longer than the containment period in some participants, continuing after day 28 in seven (47%) participants receiving candidate 1 and four (27%) participants receiving candidate 2. The last days of shedding for any of the volunteers, who were housed locally in Belgium until cessation, were day 89 in a participant receiving candidate 1 and day 48 in a participant receiving candidate 2. Shedding cessation occurred more rapidly after use of candidate 2 than candidate 1, which was complicated by the observed cessation and resumption in some participants. Shedding cessation, as defined prospectively, was met at a median of 23 days (IQR 15–36) after receiving candidate 1 and 12 days (1–23) after receiving candidate 2. Total shedding, described by the estimated median shedding index (log_10_[CCID_50_/g]), was observed to be greater with candidate 1 than candidate 2 across all participants (2·8 [95% CI 1·8–3·5] *vs* 1·0 [0·7–1·6]) and among only those shedding virus at any time (2·8 [1·8–3·5] *vs* 1·3 [0·9–2·0]) (table 3). The maximum log_10_[CCID_50_/g] observed for any participant at any time was 5·34 (day 3) in a participant receiving candidate 1, and 5·19 (day 8) in a participant receiving candidate 2. Seven participants receiving candidate 1 and one participant receiving candidate 2 had observed CCID_50_ values of more than 5·0 log_10_, but no participant maintained this concentration for more than 2 days.

**Table 3:** Stool poliovirus shedding index endpoint

	**Candidate 1 (n=15)**	**Candidate 2 (n=15)**
**Day 7**		
Number of stool samples	14	11
Number of participants shedding (%)	14 (100%)	8 (73%)
Median log_10_(CCID_50_/g) (95% CI)	4·1 (3·8–4·5)	3·3 (0·0–3·7)
Median log_10_(CCID_50_/g) among those shedding (95% CI)	4·1 (3·8–4·5)	3·4 (2·9–3·8)
**Day 14**		
Number of stool samples	13	13
Number of participants shedding (%)	10 (77%)	3 (23%)
Median log_10_(CCID_50_/g) (95% CI)	2·9 (2·8–3·3)	0·0 (0·0–0·0)
Median log_10_(CCID_50_/g) among those shedding (95% CI)	3·1 (2·9–3·6)	2·9 (2·8–4·0)
**Day 21**		
Number of stool samples	14	13
Number of participants shedding (%)	9 (64%)	3 (23%)
Median log_10_(CCID_50_/g) (95% CI)	2·9 (0·0–3·3)	0·0 (0·0–0·0)
Median log_10_(CCID_50_/g) among those shedding (95% CI)	3·0 (2·8–3·4)	3·2 (3·1–3·9)
**Day 28**		
Number of stool samples	11	8
Number of participants shedding (%)	5 (46%)	3 (38%)
Median log_10_(CCID_50_/g) (95% CI)	0·0 (0·0–2·9)	0·0 (0·0–3·2)
Median log_10_(CCID_50_/g) among those shedding (95% CI)	2·9 (2·8–3·7)	3·2 (3·0–4·1)
**Shedding index endpoint (days 7, 14, 21, and 28)**		
Number of stool samples	15	15
Median shedding index (95% CI)	2·8 (1·8–3·5)	1·0 (0·7–1·6)
Median shedding index among those shedding (95% CI)	2·8 (1·8–3·5)	1·3 (0·9–2·0)

The viral shedding index endpoint was calculated as the mean log_10_(CCID_50_/g) over days 7, 14, 21, and 28, with the lower limit of quantitation (2·75 log_10_) as an observed value and all titres in stool samples with negative shedding results set to zero. CIs were obtained via the percentile bootstrap method. If a titre was missing at days 7, 14, 21, or 28, it was replaced by a neighbouring value. If both neighbouring values were available, the titre was replaced by the mean of these values. The median shedding index among those shedding was calculated by excluding subjects that were PCR-negative for shedding at all timepoints. CCID_50_=50% cell culture infectious dose.

Most participants reported at least one mild or moderate solicited adverse event within 7 days of novel OPV2 administration: 13 (87%) participants receiving candidate 1 reported 31 events, and nine (60%) participants receiving candidate 2 reported 18 events (table 4). After receiving candidate 1, ten (67%) participants reported fatigue and eight (53%) participants reported a headache. No solicited adverse events were markedly common after participants received candidate 2; the most frequent adverse event was diarrhoea in four (27%) participants. All solicited adverse events resolved within the study period, without any permanent or long-term consequences.

**Table 4:** Solicited adverse events in the total vaccinated population

	**Candidate 1**		**Candidate 2**		**Total**	
	Number of participants (% of n=15)	Number of events	Number of participants (% of n=15)	Number of events	Number of participants (% of n=30)	Number of events
**Total**						
Solicited adverse events	13 (87%)	31	9 (60%)	18	22 (73%)	49
**Abdominal pain**						
Any	2 (13%)	3	2 (13%)	2	4 (13%)	5
Mild	2 (13%)	3	2 (13%)	2	4 (13%)	5
Moderate	0	0	0	0	0	0
**Arthralgia**						
Any	0	0	1 (7%)	1	1 (3%)	1
Mild	0	0	1 (7%)	1	1 (3%)	1
Moderate	0	0	0	0	0	0
**Diarrhoea**						
Any	1 (7%)	1	4 (27%)	4	5 (17%)	5
Mild	1 (7%)	1	4 (27%)	4	5 (17%)	5
Moderate	0	0	0	0	0	0
**Fatigue**						
Any	10 (67%)	13	2 (13%)	2	12 (40%)	15
Mild	9 (60%)	12	2 (13%)	2	11 (37%)	14
Moderate	1 (7%)	1	0	0	1 (3%)	1
**Headache**						
Any	8 (53%)	8	3 (20%)	3	11 (37%)	11
Mild	7 (47%)	7	2 (13%)	2	9 (30%)	9
Moderate	1 (7%)	1	1 (7%)	1	2 (7%)	2
**Myalgia**						
Any	3 (20%)	3	3 (20%)	3	6 (20%)	6
Mild	1 (7%)	1	3 (20%)	3	4 (13%)	4
Moderate	2 (13%)	2	0	0	2 (7%)	2
**Nausea**						
Any	2 (13%)	2	3 (20%)	3	5 (17%)	5
Mild	1 (7%)	1	3 (20%)	3	4 (13%)	4
Moderate	1 (7%)	1	0	0	1 (3%)	1
**Vomiting**						
Any	1 (7%)	1	0	0	1 (3%)	1
Mild	0	0	0	0	0	0
Moderate	1 (7%)	1	0	0	1 (3%)	1

Solicited events comprised signs and symptoms that were reported within 7 days of vaccination by use of a predefined checklist in a diary card. Participants graded their adverse events from mild to severe. No severe adverse events are reported.

All 30 participants reported at least one unsolicited adverse event, to a total of 138 events (table 2). Events were reported at similar frequencies by both groups: 72 events were reported with candidate 1 and 66 events were reported with candidate 2. 118 (86%) events were described as mild or moderate, and 67 (49%) events were considered to be either possibly or probably related to the treatment. Of the unsolicited severe adverse events, seven of the eight events (reported in six participants receiving candidate 1), and ten of the 12 events (in nine participants receiving candidate 2) were considered to be possibly related to the vaccines. All possibly related severe adverse events were abnormal laboratory findings, mainly changes in levels of alanine transaminase, aspartate transaminase, and creatine kinase. Transient increases in alaninine transaminase (≥41 U/L) were observed in three (20%) participants receiving candidate 1 and six (40%) participants receiving candidate 2. Transient increases in aspartate transaminase (≥37 U/L) were reported in five (33%) participants receiving candidate 1 and six (40%) participants receiving candidate 2. These increases peaked on days 7–14 then gradually recovered to normal values. This finding led to unplanned investigations of γ-glutamyltransferase and creatine kinase; we found no abnormal γ-glutamyltransferase or bilirubin levels, but increased creatine kinase levels in six (40%) participants receiving candidate 1 and 11 (73%) participants receiving candidate 2. Individual abnormal concentrations of creatine kinase above the normal upper limit of 190 U/L peaked at 14 632 U/L in a participant who received candidate 1 and 19 500 U/L in a participant who received candidate 2, 7 days after vaccine administration. Abnormal concentrations of creatine kinase were still present at day 28 in several participants in both groups (appendix p 1), which had returned to normal at the 42-day follow-up. We found no clinically relevant qualitative or quantitative changes in other blood chemistry or blood cell counts.

On day 0, both groups had similar titres of serum neutralising antibodies against poliovirus type-2 and all but one participant, who received candidate 2, had seroprotective titres (table 5). Increases in median titres of neutralising antibodies were observed 28 days after vaccination, with a median 8·0-fold increase after use of candidate 1 and a 12·7-fold increase after use of candidate 2, indicative of immune response to the two candidates. All participants had seroprotective titres after vaccination, with seroconversion reported in ten (83%) of 12 participants receiving candidate 1 and 11 (85%) of 13 participants receiving candidate 2 (ie, among those whose baseline titres were sufficiently below the upper limit of quantitation to allow detection of a four-fold rise).

**Table 5:** Immune responses as poliovirus type-2 neutralising antibody titres in the total study population

	**Candidate 1 (n=15)**	**Candidate 2 (n=15)**
**Geometric mean titre (95% CI)**		
Day 0	93·3 (55·3–170·0)	52·8 (25·2–123·1)
Day 28	680·9 (367·8–1098)	575·0 (330·1–871·9)
**Geometric mean fold change in titre (95% CI)**		
Day 28	7·3 (3·8–13·5)	10·9 (4·5–24·6)
**Median titre (IQR; Q1–Q3), (95% CI)**		
Day 0	56·9 (40·6–147·4; 106·7), (36·0–181·0)	36·0 (22·6–81·3; 58·6), (22·6–90·5)
Day 28	1152 (650–1448; 798), (576·0–1448)	724·1 (408·6–1152·1; 743·5), (362·0–1152)
**Median fold change in titre (IQR; Q1–Q3), (95% CI)**		
Day 28	8·00 (2·6–22·8; 20·2), (2·00–20·1)	12·73 (4·1–38·2; 34·1), (3·18–25·5)
**Seroprotection, n (%), 95% CI**		
Day 0	15 (100%), 78·2–100	14 (93%), 68·1–99·8
Day 28	15 (100%), 78·2–100	15 (100%), 78·2–100
**Seroconversion, n (%), 95% CI**		
Day 28*	10 (83%), 51·6–97·9	11 (85%), 54·6–98·1

95% CIs were calculated with the Clopper-Pearson method.

* Three participants given candidate 1 and two participants given candidate 2 had baseline titres close to the upper limit of quantitation so it was not possible to measure a 4-fold increase.

When clinical trial bulk material of viruses for candidates 1 and 2 were evaluated in the modified mouse neurovirulence assay, we did not find paralysis in any mouse inoculated with candidate 1. However, we found paralysis in four (13%) of 30 mice inoculated with candidate 2 vaccine bulk material (appendix p 2). All 15 (100%) participants receiving candidate 1 provided the necessary exploratory endpoint specimen of stool for neurovirulence assessment, which arose from day 2 to day 56 after vaccination. However, only six (40%) participants who received candidate 2 provided an exploratory endpoint specimen, of whom only two participants had shed virus samples that could be amplified to adequate concentrations to perform the neurovirulence testing; these specimens were provided on days 2 and 3 after vaccination. Among exploratory endpoint specimens from participants given candidate 1, five (33%) of 15 samples contained virus that paralysed mice: seven of 446 mice were affected, giving an overall proportion of paralysis of 2% (range 0–10 for the 15 samples). One sample from a participant given candidate 2 contained virus that paralysed four of 28 mice, giving an overall proportion of paralysis of 6·9% (0–14) across the two samples. By contrast, 70–90% of mice were paralysed by a Sabin OPV2 sample shed on day 7 in a previous trial^18^ in infants (data not shown) in the same assay, across replicates.

Among the 15 samples from participants given candidate 1, all genetic modifications engineered into candidate 1 were retained. Specifically, there were no variants consistent with reversion in domain V, the site of the main attenuation determinant in Sabin OPV2. Similarly, deep sequencing analysis of the six samples from participants given candidate 2 revealed no mutations in domain V. For both candidates, the secondary attenuation site, VP1–143, reverted in a manner consistent with Sabin OPV2, but observed changes were insufficient to cause meaningful observable paralysis in the neurovirulence test. For example, there were three samples from participants given candidate 1 in which the VP1–143 position was mutated in more than 90% of the viruses, two samples at 99%, and one sample at 93%, and none of these samples showed more than 10% paralysis.

All nasopharyngeal swabs taken on days 0, 3, 7, and the last day of containment tested negative for poliovirus by real-time RT-PCR in all participants in both groups.

## Discussion

We evaluated two novel OPV2 candidates that were designed to stabilise the poliovirus genome against acquisition of neurovirulence, to provide safer alternatives for outbreak control of cVDPVs in the era following OPV2 cessation. Our study is the first to generate human clinical data for the development of an OPV with new strains in almost 60 years. Preclinical analysis of both candidates provided strong evidence of increased genetic stability of the viral genome, with a lower risk of reversion to neurovirulence relative to Sabin OPV2 (unpublished). With the global withdrawal of OPV type-2 vaccines in May, 2016, and GAPIII containment requirements, it was determined that this initial study should be performed in fully vaccinated adults residing in a contained environment, to avoid potential environmental contamination. The successful demonstration of the candidates' safety profiles and stability in this study resulted in initiation of a larger phase 2 study in October, 2018, that is not using containment measures but that has an extensive plan for monitoring of stool samples and follow-up. This phase 2 trial in the target population will assess safety, immunogenicity, genetic stability, and neurovirulence as primary criteria for selection of candidates for further development and licensure, with secondary factors such as cost of goods sold and manufacturing yield to be considered, if necessary, to select a candidate for a phase 3 study for full licensure.

The results from our phase 1 trial indicate that both candidates are safe and immunogenic in adults. There were no serious adverse events but severe adverse events that were considered possibly to be related to the vaccines were increased blood enzyme concentrations (predominantly creatine kinase, but also alanine transaminase and aspartate transaminase), which were observed in about half the participants 1 week after vaccine administration. These increases were transient and without any abdominal symptoms or other indicators of liver damage; γ-glutamyltransferase and bilirubin concentrations were unaffected. Although we cannot fully explain these findings, they are consistent with the confined participants making unaccustomed and excessive use of the fitness equipment and daily supervised fitness training that was available in the facility. Some volunteers did report training-associated muscular pains but, for some, these reports were independent of liver enzyme increases. As observed in previous studies,^21^, ^22^, ^23^ increased creatine kinase concentrations up to 30-times the upper normal limit without changes in γ-glutamyltransferase are associated with strenuous muscular exercise, often accompanied by increases in alanine transaminase and aspartate transaminase. To assess the cause for these creatine kinase increases, group 2 (candidate 1) participants were asked to intensify their daily fitness exercises in their last week of containment, on a voluntary basis. Those who agreed were surveyed daily to recall their exercise activities, with an additional blood sample after 3 days (day 24). Results were inconclusive, with similar changes in enzyme concentrations in some but not all participants (data not shown). The subsequent larger phase 2 study with both candidates has been designed with a placebo group and participants who are not confined as in the present study, with the expectation that participants are unlikely to change their daily habits to overexercise. This phase 2 study will provide evidence as to whether the changes in enzymes were associated with the vaccination itself, since enteroviruses can cause liver enzyme increases, or the study circumstances.

Both vaccines were immunogenic in the IPV-only vaccinated study population, with substantial increases in type-2 neutralising antibody titres 4 weeks after vaccination, and showed evidence of intestinal replication, evidenced by stool shedding. We did not find any evidence of nasopharyngeal shedding from any participant. This finding is reassuring because procedures for collection and analysis of nasopharyngeal samples were as rigorous as for the stool samples. As discussed by Hird and Grassly,^24^ nasopharyngeal shedding has not been extensively studied and previous reports of decreased nasopharyngeal shedding of wild-type virus in IPV-vaccinated children are confounded by variations in age and other factors. We observed differences in stool shedding between the candidates, both in duration and magnitude of faecal titres, with observed shedding greater for candidate 1 than candidate 2. Shedding of both viruses persisted for longer than the study containment period in seven (47%) participants given candidate 1 and four (27%) participants given candidate 2. We observed no laboratory or immunological abnormalities in participants who shed for the longer periods. Although we have no comparative data for monovalent OPV2 in an IPV-only vaccinated adult population, in IPV-only vaccinated infants challenged with monovalent OPV2, the extent of viral shedding measured by the shedding index was similar to, or greater than, the levels we observed; a substantial proportion of infants (around 25%) had a median viral shedding index score of more than 5·0 log_10_, a value that was rarely observed for individual samples in our study.^20^ This result suggests that viral shedding might not be substantially increased and could even be lower with these novel candidates compared with vaccines containing Sabin-2, which will require additional evaluations in the target population for confirmation.

Although there was evidence of accumulating genetic substitutions in shed virus for both candidates, as would be anticipated for an RNA virus, no variants consistent with increased virulence were detected in domain V of the 5ʹ-untranslated region, the site of the primary determinant of attenuation for Sabin OPV2 (nucleotide 481). Notably, in both candidates, the unprotected secondary attenuation site, VP1–143, reverted in a manner consistent with expectations for Sabin OPV2. However, shed virus with variants in this position showed low paralysis (1·6–6·9%), compared with a sample of reverted Sabin monovalent OPV2 (90%) in the same test. These data are consistent with previous values for monovalent OPV2 and provide strong support for the improved genetic and phenotypic stability of the novel OPV2 candidates.^25^, ^26^

In one clinical study,^27^ all type-2 virus that was shed 7 days after administration contained substantial (33–96%) reversion at the primary attenuation site (nucleotide 481); another study^28^ showed similar results, with almost 100% reversion within 14 days after trivalent OPV vaccination. These neurovirulence data provide encouraging evidence for the genetic and phenotypic stability of both candidates relative to the rapid loss of attenuation of Sabin OPV2 vaccines that result in high paralysis rates in transgenic mouse assays.^15^ Therefore, irrespective of other variations occurring over time, the excreted viruses were expected to maintain their attenuation to a large degree.

Limitations of this phase 1 study were that it necessarily involved few participants, used what is expected to be the maximal dose of vaccine virus and, because of the global cessation of elective use of monovalent OPV2, there was no direct comparator to test both candidates.

Our study shows that these two novel OPV2 candidates have the potential to provide more genetically stable alternatives to the current Sabin monovalent OPV2 that is stockpiled for control of cVDPV2 outbreaks. With all live type-2 polioviruses withdrawn from routine immunisation use, the global population is now reliant on IPV to provide immunity against type-2 polioviruses. The limited primary intestinal immunity provided by IPV requires that an outbreak response must rely on Sabin oral live-attenuated poliovirus vaccines to interrupt person-to-person transmission.^16^, ^29^ With the known reversion of Sabin monovalent OPV2 and developing evidence that it might have generated new cVDPVs in some settings,^7^ novel candidate vaccines such as those we have tested could be crucial components of efforts to ensure complete and permanent global eradication of poliovirus type-2.

For **data access** email openaccess@gatesfoundation.org

## Data sharing

The data are not publicly available because of ethical restrictions on participant privacy, but data for this study will be made available to others in the scientific community upon request after the ongoing phase 2 study of the two vaccine candidates has been completed and the scientific data from the development of the two candidates have been fully published. Standard criteria for making data available for valid research projects will be used, following application by suitably qualified researchers. For data access, please contact the Gates Foundation.
