# Supplementary material for: The safety and immunogenicity of two novel live attenuated monovalent (serotype 2) oral poliovirus vaccines in healthy adults: a double-blind, single-centre phase 1 study
Source: Lancet. 2019 Jul 13;394(10193):148–58. doi: 10.1016/S0140-6736(19)31279-6 (PMC6626986; doi:10.1016/S0140-6736(19)31279-6)

THE LANCET

## Supplementary appendix

This appendix formed part of the original submission and has been peer reviewed. We post it as supplied by the authors.

Supplement to: Van Damme P, De Coster I, Bandyopadhyay AS, et al. The safety and immunogenicity of two novel live attenuated monovalent (serotype 2) oral poliovirus vaccines in healthy adults: a double-blind, single-centre phase 1 study. *Lancet* 2019; published online June 4. http://dx.doi.org/10.1016/S0140-6736(19)31279-6.

**Supplementary Figures.**

1. Group creatine kinase levels over the 28-day containment period.
2. Proportions of EES samples causing mouse paralysis.

**Supplementary Figure 1.**

Creatine kinase levels. Values show mean levels grouped according to being in the normal range (closed symbols) and abnormal ranges (open symbols) for candidate 1 (orange) and candidate 2 (blue). Error bars show ranges of values for each group.

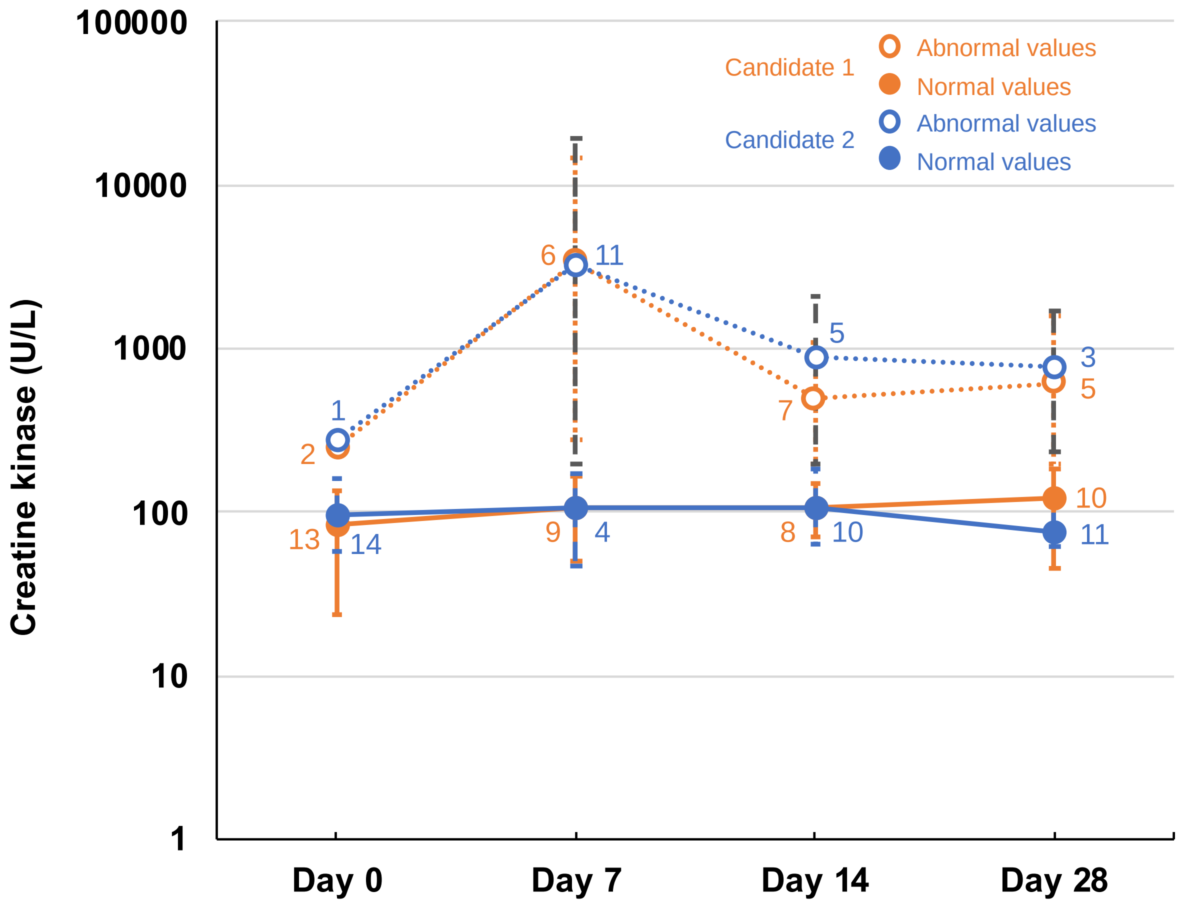

**Supplementary Figure 2.**

Mouse paralysis proportion for Exploratory Endpoint Samples (EES), as well as clinical trial material (“Vaccine”) in a single-dose (4.0 $\log_{10}$ [$CCID_{50}$] intraspinal inoculum) transgenic mouse model. Ten mice per sample were assayed, alongside controls, with three replicates each. Points indicate samples/subjects (combined over each replicate), with diamonds indicating the overall means and horizontal lines indicating the median across subjects

* Reference range of 70–90% paralysis developed from repeated assay (n=5) of a type-2-containing sample from an infant vaccinee who received mOPV2 at 40 weeks, following bOPV at 6/10/14 weeks and IPV at 14/36 weeks in a prior clinical trial.[21] Sample collected 7 days post-challenge and selected based on high reversion (89% 481G).

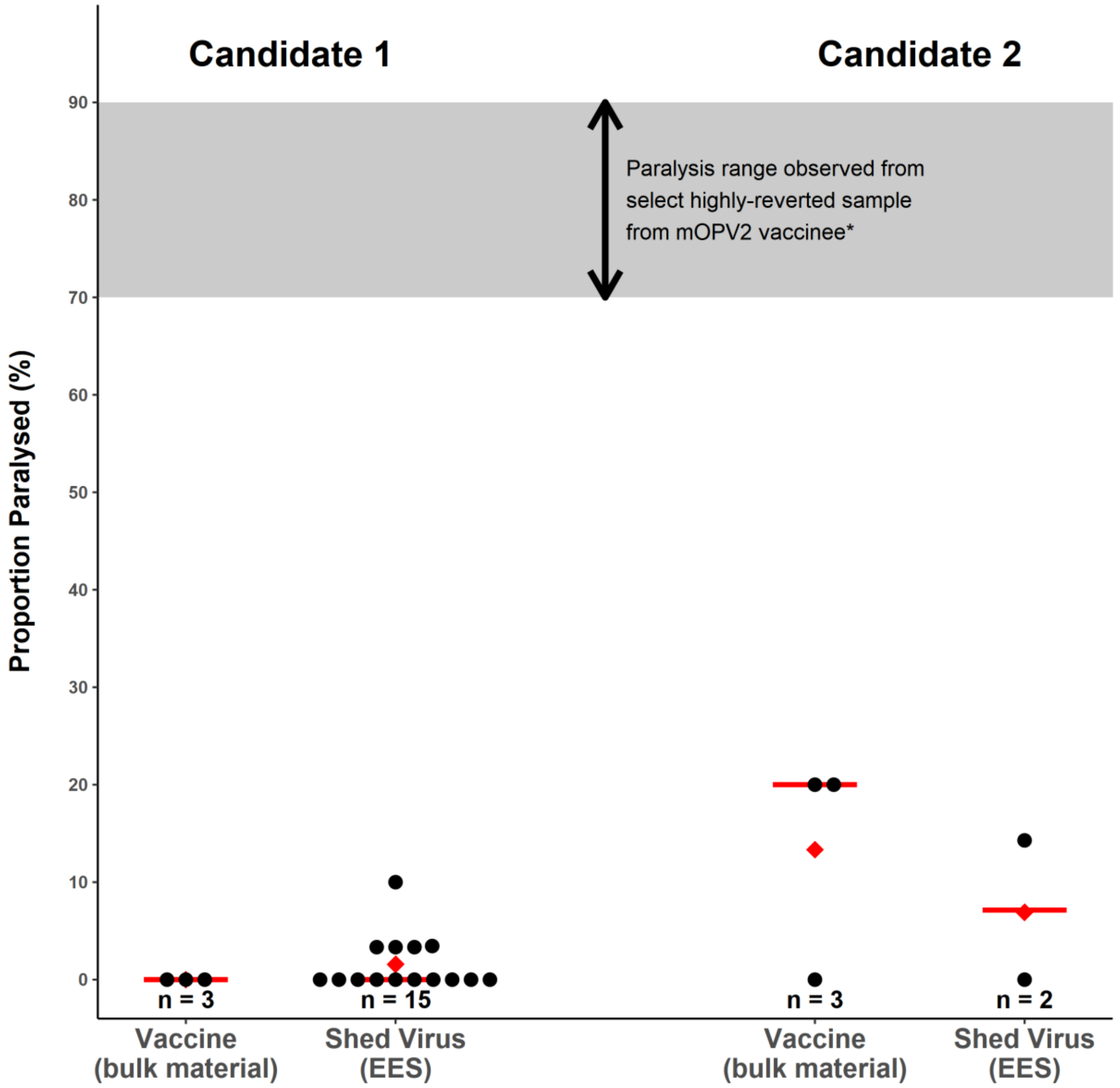

Supplement: Supplementary appendix [file mmc1.pdf]
